# Developing the Accuracy of Vital Sign Measurements Using the Lifelight Software Application in Comparison to Standard of Care Methods: Observational Study Protocol

**DOI:** 10.2196/14326

**Published:** 2021-01-28

**Authors:** Thomas L Jones, Emily Heiden, Felicity Mitchell, Carole Fogg, Sharon McCready, Laurence Pearce, Melissa Kapoor, Paul Bassett, Anoop J Chauhan

**Affiliations:** 1 Portsmouth Hospitals NHS Trust Portsmouth United Kingdom; 2 Xim Catalyst Centre Southampton Science Park Chilworth United Kingdom; 3 Mind over Matter Medtech Ltd Tonbridge United Kingdom; 4 Statsconsultancy Ltd Amersham United Kingdom

**Keywords:** health technology, remote monitoring, vital signs, patient deterioration

## Abstract

**Background:**

Vital sign measurements are an integral component of clinical care, but current challenges with the accuracy and timeliness of patient observations can impact appropriate clinical decision making. Advanced technologies using techniques such as photoplethysmography have the potential to automate noncontact physiological monitoring and recording, improving the quality and accessibility of this essential clinical information.

**Objective:**

In this study, we aim to develop the algorithm used in the Lifelight software application and improve the accuracy of its estimated heart rate, respiratory rate, oxygen saturation, and blood pressure measurements.

**Methods:**

This preliminary study will compare measurements predicted by the Lifelight software with standard of care measurements for an estimated population sample of 2000 inpatients, outpatients, and healthy people attending a large acute hospital. Both training datasets and validation datasets will be analyzed to assess the degree of correspondence between the vital sign measurements predicted by the Lifelight software and the direct physiological measurements taken using standard of care methods. Subgroup analyses will explore how the performance of the algorithm varies with particular patient characteristics, including age, sex, health condition, and medication.

**Results:**

Recruitment of participants to this study began in July 2018, and data collection will continue for a planned study period of 12 months.

**Conclusions:**

Digital health technology is a rapidly evolving area for health and social care. Following this initial exploratory study to develop and refine the Lifelight software application, subsequent work will evaluate its performance across a range of health characteristics, and extended validation trials will support its pathway to registration as a medical device. Innovations in health technology such as this may provide valuable opportunities for increasing the efficiency and accessibility of vital sign measurements and improve health care services on a large scale across multiple health and care settings.

**International Registered Report Identifier (IRRID):**

DERR1-10.2196/14326

## Introduction

### Background

Vital sign measurements—also referred to as patient observations—are a widely adopted and integral component of clinical care. They involve the intermittent observation and measurement of a person’s basic body functions. Traditionally, vital signs have included blood pressure, temperature, pulse rate, and respiratory rate measurements, although a number of other parameters are now also included. Changes in vital sign measurements may be the first and earliest indication of an abnormal physiological change in a patient, offering a source of essential information to the assessing clinician.

Within the hospital setting, and especially in high-dependency areas, vital sign measurements are undertaken regularly to support the recognition of clinical deterioration and the need for intervention and escalation of care; often, these measures are used within a multiparameter, early warning, scoring system [1,2]. In primary care services, vital signs measurements are similarly important, for example, in the assessment of infants and children [3] and in those with long-term conditions, while in community and residential care settings, home measurement of vital signs can be particularly useful in monitoring the health status of an increasingly aging population.

### Challenges, Gaps, and Inefficiencies

Despite their importance for clinical decision making, the accuracy and timeliness of vital sign observations is a recognized area in need of improvement [4-6]. There are several reasons behind the current challenges with vital sign measurements. First, a number of different methods of measurement of vital signs is available, both invasive and noninvasive, and a minimum level of training, skill, and competence is required by operators. A lack of standardized technique and the potential for operational errors can negatively affect measurement accuracy [7,8]. Second, the time required to take vital sign measurements can impact the frequency and quality of assessments, especially in an environment already experiencing a heavy workload and staff shortages. There is some evidence that complacency can develop when vital sign measurements become perceived as low-priority tasks [9]. Third, the quality of recording of vital sign measurements, including the accuracy and completeness of documentation, as well as the way in which this information is viewed in a clinical record, can all influence clinical management decisions [10]. Improvements in recording was one of the recommendations from the enquiry into poor care and high mortality rates among patients at the Mid-Staffordshire NHS Foundation Trust [11]: “The recording of routine observations on the ward should, where possible, be done automatically as they are taken, with results being immediately accessible to all staff electronically.”

Current standard of care methods for measuring vital signs can also be inconvenient and uncomfortable for patients. Most noninvasive techniques will still involve some form of contact, which can cause skin irritation [12]. Monitoring equipment can create hazards and interfere with patient care, mobility, and sleep, while its conspicuousness can be emotionally upsetting, for example to visitors in critical care settings [13,14]. Stress or anxiety arising from the observation process itself can also result in misleading measurements, which may not be representative of the patient’s clinical state; for example, the “white coat effect” may lead to raised blood pressure readings during clinical assessments despite normal blood pressure in other settings [15].

### Proposed Innovation and Delivery

These identified challenges have led to significant research into the role that advanced technologies may play in improving the quality, timeliness, and accuracy of vital sign measurements. These advanced techniques have included using thermal imaging analysis [16,17], Doppler effect observations [18,19], and photoplethysmography [20]. There has been particularly rapid development in the use of image-based or video-based monitoring over the last decade [21] in a bid to improve the quality and accessibility of physiological monitoring.

Photoplethysmography is an optical measurement technique that uses changes in properties of the skin to detect blood volume changes in the microvascular tissue bed [22]. Photoplethysmography was originally shown to operate at red and near infrared wavelength but has since been found to also work effectively using ambient light as the illumination source [23]. Changes in the light reflected from the skin surface due to volumetric changes in the facial blood vessels can detect small variations in perfusion, providing valuable information about the cardiovascular system [24]. Photoplethysmography is now used to assess a number of physiological parameters, including heart rate, oxygen saturation [25], blood pressure [26], and respiratory rate [27,28]

**Figure 1:**
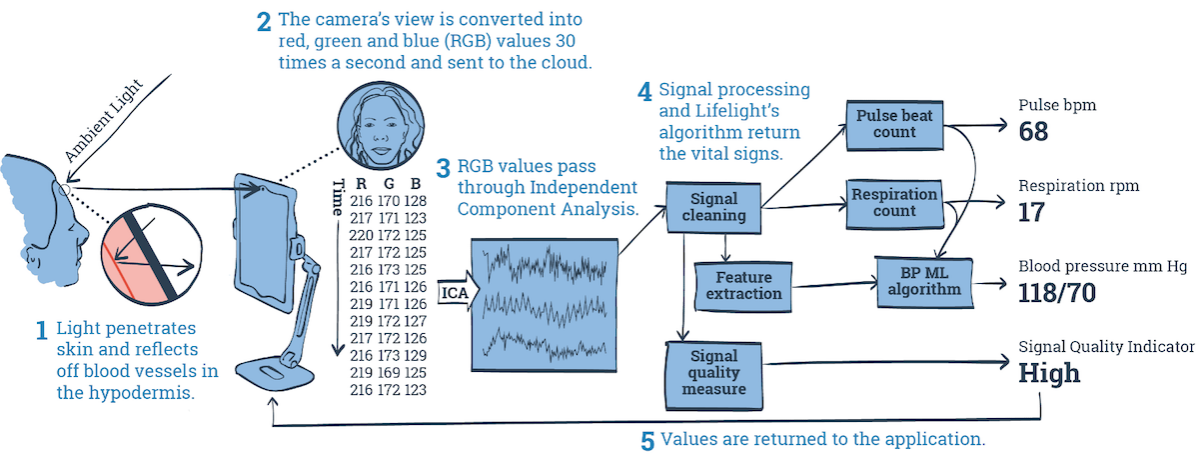
Representation of the use of photoplethysmography in the Lifelight software application. BP: blood pressure; ICA: independent component analysis; ML: machine learning.

**Figure 2:**
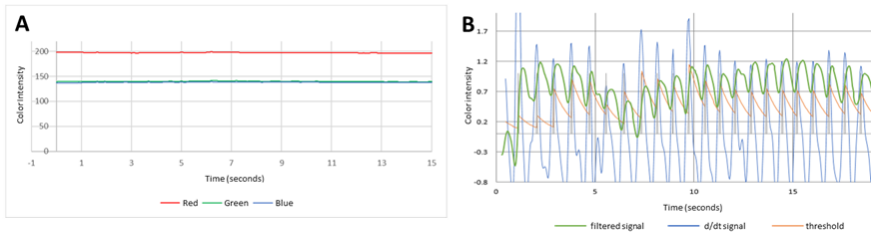
Chart of the (A) raw remote photoplethysmography (rPPG) signal received by the Lifelight algorithm and (B) some of the intermediate signal traces derived from that raw rPPG signal. These processed signals are used in turn to derive vital signs—in this example, pulse.

Lifelight is a software application that, when installed on a laptop, mobile device, or smartphone with an integral camera, uses remote photoplethysmography (rPPG) and live video capture to calculate these 4 most important physiological parameters (Figure 1). The application captures the average color of an area of the face (the “region of interest”) and sends this as red, green, and blue (RGB) color values to the server for further processing. The RGB data are recorded 30 times every second for 60 seconds until a full set of 1800 RGB points has been delivered. Having eliminated artefacts and interference, the application can derive a plethysmographic signal after 30-60 seconds of data collection (Figure 2). The strength of the pulsatile component of the raw video signal generated by the app is small (approximately 1%-2% of the signal). Highly tuned signal processing techniques are used to derive a pulse waveform that is further processed and counted to give heart rate, respiratory rate, and through comparison of color channel ratios, oxygen saturation. The shape of the pulse waveform undergoes additional analysis to derive features that describe the pulse’s morphology, and the resulting morphological feature set is fed into an algorithmic model derived through machine learning to predict systolic and diastolic blood pressures. This technology has the potential to take noncontact measurements with a device that has minimal requirements for clinical skill or training.

During this preliminary study, measurements predicted by the Lifelight software will be compared with standard of care measurements for a large population sample of inpatients, outpatients, and healthy people. The study will create a dataset for use in developing and testing the accuracy of the software. Predicted vital signs from recorded rPPG signals will be evaluated against measured vital sign values, and the success of changes to the underlying algorithm to improve the predictions will be gauged. This study will also explore how the performance of the algorithm varies with some patient characteristics including health condition and medication.

## Methods

### Aims of the Study

The overall aim of this study is to improve the accuracy of the estimated heart rate, respiratory rate, oxygen saturation, and blood pressure measurements predicted by the Lifelight software application through development of its algorithms. The primary objective is to compare the estimated Lifelight measurements with those from standard of care measurements. The secondary objective is to explore the impact of a range of variables (including age, sex, temperature, health condition, medication, skin tone, and ambient lighting) on the accuracy of the heart rate, respiratory rate, oxygen saturation, and blood pressure estimates made by the algorithm.

### Participants and Recruitment

Study participants will be recruited from a single study site in the United Kingdom (Queen Alexandra Hospital in Portsmouth Hospitals NHS Trust). Both adults and children will be included in the study population. Participants will include people who are attending the hospital, as an inpatient, an outpatient, a friend or relative of a patient, a visitor, or a member of hospital staff. Inpatients and outpatients will be approached about potential participation by study staff during their stay or while waiting for an appointment; in the case of pediatric potential participants, parents or guardians will be approached. Members of the public will be recruited through recruitment posters and information stands at public sites in the hospital. Members of the study team will be available to discuss the study with any interested individuals who approach the information stands or email or phone study staff. Hospital staff will be recruited through email recruitment campaigns, discussion in team meetings, posters in staff areas, and information stands.

Inclusion criteria include age over 3 years, sufficiently conversant in English language, and able and willing to comply with all study requirements and to provide written informed consent (either themselves or empowered by law to provide it). There are no exclusion criteria. Eligible potential participants interested in the study will receive written and verbal information on the study aims, what is involved, and the requirements for participation, including a participant information sheet; in the case of pediatric potential participants, age-appropriate information will be provided to the child, and a parent information sheet will be available for their parents or guardians. Participation in the study will be entirely voluntary, refusal to participate will involve no penalty nor loss of medical benefits, and participants may withdraw from the study at any time.

An estimated sample size of 2000 participants is planned to generate measurements for use in the training dataset. The final sample size of the study will depend upon the incremental improvement in accuracy of the Lifelight system, and the algorithm development cannot be predicted prior to enrollment. The intended study sample will be selected through cluster sampling of the hospital population, including approximately equal proportions of inpatients, outpatients, and healthy control groups. The study sample will aim to capture a locally representative cohort regarding age, sex, health condition, and skin tone. Approximately 25% of participants will be children between 3 and 16 years old. Vital sign measurements from an additional 1000 patients will be taken at the end of the study period to be used for a validation dataset.

### Study Design

**Figure 3:**
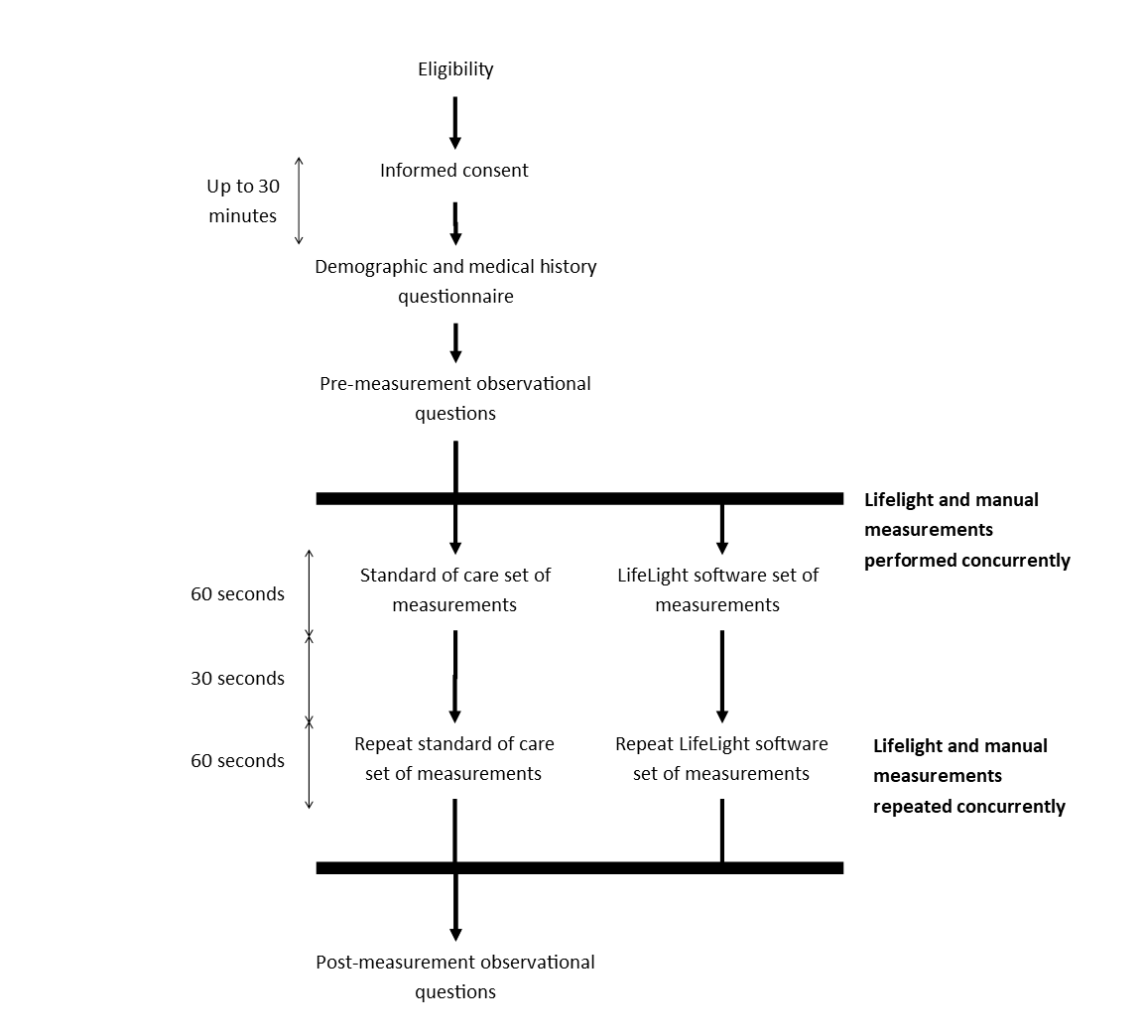
Study flow diagram for VISION-D study participation.

The study will adopt a prospective, observational design (Figure 3). The study will collect both training and validation datasets of vital sign measurements, which will be compared with the algorithm’s estimated measurements.

### Data Collection and Outcome Measures

#### Measurements

Participation in the study will involve 2 methods of measuring 4 specific vital signs: heart rate, respiratory rate, oxygen saturation, and blood pressure measurements. One method of measurement will be through manual direct physiological measurement of vital signs, while the other will be calculation of vital sign measurements from digitally captured changes in reflected light from the skin surface. Both sets of measurements will be taken concurrently by 2 members of staff during the same 60-second measurement period. Both Lifelight and manual measurements will then be repeated following the initial observations, yielding a total of 4 sets of measurements for each participant. Once all measurements have been taken and recorded, the study staff member will complete the postmeasurement observation questions and record all answers manually on paper case report forms.

#### Data Collection

Data collection will be face-to-face by trained study staff members. All measurements will be taken by adult or pediatric nursing staff, assisted by clinical trial assistants. All staff will receive appropriate competency-based training in the set-up, maintenance, and limitations before utilizing any of the medical devices required for this study, including the Lifelight software and iPad device; all staff involved in data collection will also complete Good Clinical Practice training.

#### Premeasurement Data Collection

**Figure 4:**
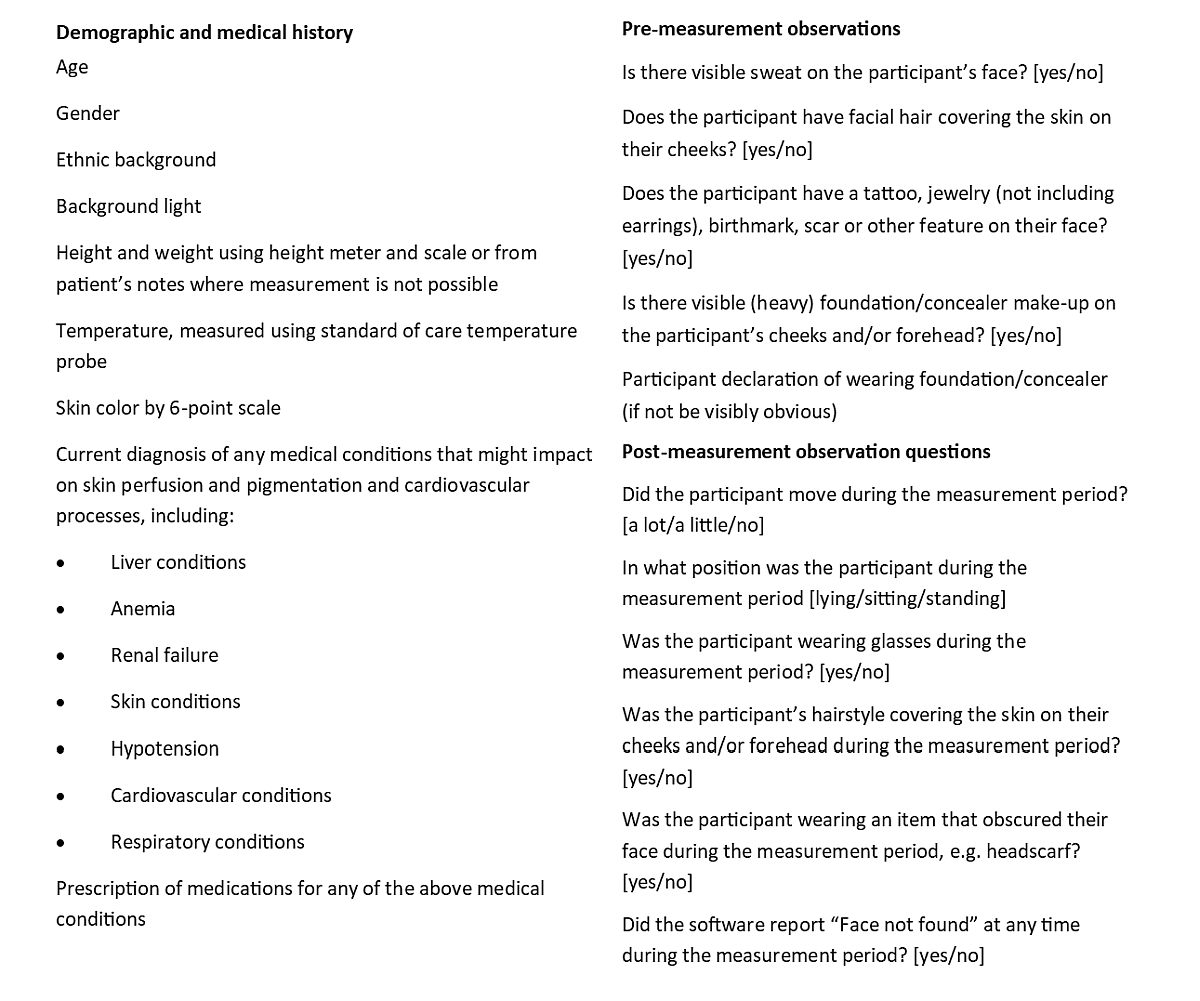
Observations and questions used for data collection during the study.

Prior to any measurements being taken, the study staff member will complete a very brief set of demographic and medical history questions and a set of premeasurement observations with the participant. All answers will be recorded manually on paper case report forms; the information gathered is detailed in Figure 4.

#### Standard of Care Manual Measurements

The direct physiological measurement of vital signs will be taken manually using instruments normally employed during standard care. The following methods, which are already currently in routine use at the study site and are widely accepted as standard practice, will be adopted for vital sign measurements in this study.

The Welch Allyn Vital Signs Monitor device will be used to determine heart rate (pulse) measurements (primarily from the oxygen saturation measurement methodology or, if unavailable, from the blood pressure measurement method).

Respiratory rate will be determined from manual counting of observed inspirations during the 60-second measurement period.

A standard clinical finger clip sensor (as part of the Welch Allyn Vital Signs Monitor device) will be used for oxygen saturation measurements. The average oxygen saturation measurement over the 60-second measurement period will be used as the final result, ensuring that the oximeter is picking up an appropriate waveform.

A standard clinical automatic sphygmomanometer with appropriately sized cuff (width at least two-thirds of upper arm length) on the participant’s upper arm (on the side opposite the finger clip sensor) will be used for blood pressure measurements (as part of the Welch Allyn Vital Signs Monitor device). This will be operated at the start of the 60-second measurement period.

All equipment used in the study for direct manual vital signs measurements is newly purchased, is dedicated for study use, and has been serviced and calibrated in line with manufacturers’ instructions. All medical devices have a CE marking, have been locally approved for use in the hospital setting, and have been cleaned in accordance with local infection control guidelines. The same protocol for measurement will be adopted for all direct manual measurements, to ensure standardization of data collection. The stopwatch integrated within the Lifelight software application will be used to identify the beginning and end of the 60-second measurement period and to ensure that the measurements are taken exactly concurrently; this will be announced by the study nurse so that both the members of staff and the participant are aware of the measurement period. The results are automatically displayed on the Welch Allyn Vital Signs Monitor device and will be recorded manually at the time of measurement onto paper case report forms, before being later transcribed into a study-specific database, anonymized, and sent electronically to the Lifelight developer. For inpatients, these measurements will also be recorded in their electronic observations record, used by the clinical team responsible for their care; other participants, whose observations are outside of normal ranges and clinically significant, will be referred to their general practitioner or to an urgent care provider, as necessary, for further investigation (Table 1).

**Table 1:** Normal measurement ranges and ranges requiring referral for further investigation (adapted from ranges for physiological parameters as detailed in the Royal College of Physicians’ National Early Warning Score).

Physiological parameter	Description	Normal range for adults	Range requiring referral to GP^a^ for adults	Range requiring referral to urgent care for adults
Heart rate	Pulse rate or number of heart beats per minute	51-90 bpm	N/A^b^	<40 bpm, >120 bpm, or irregular
Systolic blood pressure	The pressure of circulating blood on the walls of blood vessels	111-219 mm Hg	>160 mm Hg	<90 mm Hg or >180 mm Hg
Oxygen saturation	The oxygenation of fresh arterial blood	≥96%	N/A	<92%
Respiratory rate	Breathing rate or breathing interval	12-20 breaths/minute	N/A	N/A

^a^GP: general practitioner.

^b^N/A: not applicable.

#### Using the Lifelight Software

The digital measurement of changes in reflected light from the skin surface will be made using the Lifelight software on an Apple iPad (6^th^ generation Wi-Fi, 32 GB, 9.7 inch [diagonal] display) mobile tablet device. Background light intensity will be determined using a handheld illumination, or lux, meter (URCERI MT-912 Light Meter) to measure the density of light in the area near the participant’s face. Changes to the environment will then be made as necessary to ensure that the camera view of the participant is well illuminated, for example by turning on more lights or opening window blinds. The Lifelight software includes a face-tracking algorithm, which will detect the presence of a human face in the image and select a rectangular region of interest (usually on the forehead). Stepwise instructions from the Lifelight software will guide the operator and automatically start and stop the measurements, which will be taken throughout the 60-second measurement period. The measurements taken by the Lifelight software will not be displayed on the iPad screen, but will be recorded and stored locally on the mobile tablet device until it has internet connectivity, at which point anonymized data will be uploaded to the Lifelight developer’s secure database. This will ensure that measurements will not be revealed to either the study staff or the participant, thereby reducing the risk of confounding.

The same protocol will be adopted for all measurements taken with the Lifelight software, to ensure standardization of data collection; in particular [29,30] the mobile tablet device will be held as still as possible, at a distance of approximately 1 meter away from the participant and angled towards their face. Participants will be requested to keep as still as possible during the measurement period and to refrain from moving their head, talking, or chewing. Participants will be requested to remove any garments (eg, hats, scarves) and arrange hair (including bangs) so that facial skin is not grossly obscured and the facial skin surface area is maximized. This excludes instances where garments or other features are of a sensitive nature (eg, worn for religious purposes). Participants will be repositioned as necessary to ensure adequate lighting to illuminate the facial skin and that there are no other people (including study nurses or assistants) coming into the line of sight of the device camera.

When these conditions are not achieved during the trial, because it is impractical to do so or because of participant nonconcordance, the study staff member will indicate so in their answers to the postmeasurement observation questions.

### Privacy and Data Protection

A unique sequential patient identifier will be generated for each participant in the study, and no identifiable data will be included in the stored data. All documents will be stored securely and only accessible by study staff and authorized personnel. All transmitted data will be encrypted before sharing and stored in the developer’s secure database.

Fully pixelated images without any video data will be uploaded during the study as default for both adult and pediatric participants. However, an option is available within the adult consent form for participants to increase the level of video data collected, sharing either data with identifying features obscured or sharing full face video if they wish.

### Data Analyses

Statistical analyses will use the Deming regression to assess the degree of correspondence between the vital sign measurements predicted by the Lifelight software and the direct physiological measurements taken manually using “gold standard” methods. Subgroup analyses will determine variability in the degree of correspondence for patient characteristics (specifically age, sex, BMI, temperature, health condition, medication, skin tone, ambient lighting, presence of visible sweat, and a 3-way comparison of confounding factors [cosmetics/moisturizer, presence of facial scars/tattoos, and none]). Subgroup analyses will also be carried out for participants with readings outside of normal clinical ranges (Table 1), as well as for groups formed according to measured values. The impact of any changes made to improve the performance of the algorithm will be analyzed by stratification of the data by each algorithm version. No changes will be made to the algorithm during analysis of the validation dataset.

All values will be log transformed for analysis. All statistical analyses will use r (version 3.5.0) via RStudio for Windows [31]. The total least squares approach of the Deming regression will be implemented via the pca functionality of r with appropriate corrections to account for the multiple sets of readings per participant.

Continuous variables will be categorized to allow comparison. BMI will be derived from height and weight measurements and separated into categories of underweight, normal, overweight, and obese. Categories will be based on internationally accepted classification for adults [32] and age-specific and sex-specific classifications for children [33].

All analyses will be completed per protocol, since there is no intention to treat. There will be no imputation of missing or implausible data, and any missing, implausible, or problematic readings will be excluded from analysis. Where height and weight data are not available, blood pressure will not be estimated. If the Lifelight software is unable to detect the participant’s face during the measurement period, this will be recorded in the case report form, and the measurements will be deleted from the dataset.

## Results

The prototype Lifelight technology has been in development over the past 5 years. Recruitment of participants to this study began in July 2018, and data collection will continue for a planned trial period of 12 months.

## Discussion

Digital health technology is a rapidly evolving area for health and social care. The monitoring of vital signs to provide early warning of critical health events is one area of clinical practice that is benefiting from the digital transformation currently being experienced across health care services, and in particular, image-based monitoring methods are undergoing significant research and development. For example, Shao et al [34] presented a noncontact method to monitor blood oxygen saturation, which gave results consistent with those measured using a reference contact oxygen saturation device, while Jain et al [35] used face video–based photoplethysmography to predict blood pressure and heart rate. A recent systematic review of image-based, noncontact methods of monitoring heart rate, blood pressure, respiratory rate, and oxygen saturation analyzed over 160 studies. However, 76% of these included 20 or fewer subjects, and only 20 of these studies were carried out in clinical (rather than academic) settings [36]. Use of the Lifelight software application for noncontact remote monitoring may provide an opportunity for increasing the efficiency and accessibility of vital sign measurements on a large scale across multiple health and care settings, and studying its performance in a very large cohort within a clinical environment is a crucial area of development.

There are some anticipated limitations that the study may encounter. Movement of the subjects can create noise in the readings, and technical requirements such as internet access and lighting are required for the application. Ensuring that a locally representative cohort of participants is involved in the study may also be challenging, and regular interim review of recruitment and targeted stratified sampling will therefore be carried out as necessary.

Anecdotal feedback during the design and development of this study has been very positive about the potential of this novel approach to patient observations, particularly its simplicity. However, concerns have been raised around how remote monitoring of vital signs might impact the interaction between patients and health care professionals, in particular nurses, and the risk it may present of missing other deterioration cues, thereby compromising the overall quality of the patient assessment. This will be an important area of research for future studies.

This initial exploratory study will enable the software application to be further developed as a preventative monitoring technology. Subsequent work will evaluate the performance of the application in healthy participants subjected to physiological perturbations and in participants with a range of health characteristics. The results of this study, alongside other extended validation trials, will be used to support the technology to gain CE marking. By demonstrating that the device is fit for its intended purpose and meets safety legislation requirements, the Lifelight software will become available for use across Europe as a medical device.

## Appendix

Multimedia Appendix 1 Evidence of grant funding awarded.

Multimedia Appendix 2 Peer review completed and submitted to received Health Research Authority approvals to carry out the study in a UK NHS setting.

## Abbreviations

RGB: red, green, blue
rPPG: remote photoplethysmography
