# Supplementary material for: Developing the Accuracy of Vital Sign Measurements Using the Lifelight Software Application in Comparison to Standard of Care Methods: Observational Study Protocol
Source: JMIR Res Protoc. 2021 Jan 28;10(1):e14326. doi: 10.2196/14326 (PMC7878110; doi:10.2196/14326)
Supplement: Multimedia Appendix 1 [file resprot_v10i1e14326_app1.pdf]

From: **Penny Richold** penny.richold@hee.co.uk
Subject: SBRI Phase 2 Interview Details
Date: 24 August 2017 at 16:00
To: laurence@xim.ai

PR

Dear Laurence Pearce,
We can confirm that your SBRI Healthcare Phase 2 interview regarding **'ViVa - Visual Vitals passive vital signs measurement in primary care'** in the **'General Practice - Workload and Demand Management'** competition will take place:
Date: Wednesday 1st Nobember
Venue: ABHI, 107 Grays Inn Rd, London WC1X 8TZ
Time : 12:00
The format of the Phase 2 interview will be very similar to the format you will be familiar with from your experience of the Phase 1 process. However, this time each project is allowed a maximum of four presenters and the interview will last a total of 50 minutes. Presenters will have up to 18 minutes to describe:

- Your project's progress in Phase 1;
- Plans for Phase 2 and beyond, including what stage of development the product is likely to have reached by the end of Phase 2;
- The current state of the developed technology, including the potential clinical and commercial impact of the product.

The remainder of the interview time will be taken up with a question and answer session with the interviewing panel.
The interviewing panels will, as was the case in Phase 1, be composed of clinical, commercial and technical experts, and some will have participated in the Phase 1 interviews. While panels will have been supplied with your project's Phase 1 final report, Phase 2 application and the health economics report, they will expect companies to be able to fully answer detailed questions about the contents of any of these documents.
PowerPoint presentations can be used and if your project has a video, that can provide details on the current state of your project's technology, then please do consider using it, if appropriate, and embed this in your presentation, as it will save time. Presenters are also encouraged to bring prototype devices for the panel to view, where this is appropriate. **Please do not rely on the availability of Wi-Fi or any internet connectivity.**
Any videos or PowerPoint presentations that groups would like to use for their interview need to be sent to the SBRI management team by 1pm on the last working day before their scheduled interview day. Files should be emailed to: sbrienquries@hee.co.uk. All files sent should be compatible with Windows Microsoft Office release 2003 or later. If videos are embedded in your PowerPoint presentation, please let us know their location so we can test them for you in advance.
I regret to say that your interview cannot be moved or rearranged in any way.
Please confirm your attendance as soon as possible and if you have any questions regarding your Phase 2 interview, please let me know.

Penny Richold
Administration Manager
Health Enterprise East Ltd
Milton Hall
Ely Road
Milton
Cambridge
CB24 6WZ

T: 01223 928040
Penny.Richold@hee.co.uk

From: **Chris J. Warwick** Chris.Warwick@hee.co.uk
Subject: RE: SBRI Phase 2 Interview Details
Date: 4 April 2019 at 10:31
To: Laurence Pearce laurence@xim.ai, Gina Pelletier gina@xim.ai
Cc: Penny Richold penny.richold@hee.co.uk

CW

Hi Laurence,

The panel members for your Phase 2 interview are below:

**Anne Blackwood**	CEO, Health Enterprise East Ltd
**Hein Leroux**	Primary Care, GP Lead
**Dr Alison Tavaré**	Bristol GP and GP advisor West of England AHSN
**Tim Meehan**	Tech Assessor - Director at Horizon SciTech
**James Barsby**	Alliance Manager, West of England AHSN
**Chris Sawyer**	Innovate UK
**Emily Boyce**	Innovation Manager, Health Enterprise East Ltd

Best Regards,
Chris

mailto:Pearcelaurence

Dr Chris Warwick
Medtech Consultant & SBRI Healthcare Programme Manager
Health Enterprise East Ltd.
Milton Hall, Ely Road, Milton, Cambridge, CB24 6WZ
Tel: 01223 928040 **Mobile**: 07971 560926
www.hee.co.uk - www.sbrihealthcare.co.uk

Health Enterprise East
Realising innovation

From: **Emily C. Boyce** emily.boyce@hee.co.uk
Subject: SBRI Healthcare: Phase 2 Provisional Award
Date: 24 November 2017 at 16:15
To: Laurence Pearce laurence@xim.ai
Cc: SBRIEnquiries SBRIEnquiries@hee.co.uk

EB

Dear Laurence,

We are pleased to confirm that the SBRI Healthcare panel in the 'Workload and Demand Management' category of the 'GP of the Future' competition have recommended awarding a SBRI Healthcare Phase 2 contract to xim Ltd, to support the project entitled "ViVa - Visual Vitals passive vital signs measurement in primary care". This SBRI Healthcare award will have a total value not exceeding £850,000 inclusive of VAT.

As discussed in your call with the Joop, we are currently working with NHS England to achieve ratification of these Phase 2 awards. We have a strong expectation that all awards will shortly be officially confirmed, however before this occurs we would ask that this communication is considered in confidence, and as previously with the Phase 1 contracts, no PR should be released before SBRI Healthcare publicly announces the awards.

As the awarded amount is lower than that applied for in the company's original Phase 2 application we appreciate that some activities included in the company's original Phase 2 application may need to be amended. We would therefore a revised Phase 2 proposal that includes a summary of the project's activities, milestones and financials that is in line with the awarded amount, as laid out according to the format provided in the attached Word and Excel templates. Due to time restrictions we are asking that this revised proposal is supplied to the SBRI Healthcare team at the latest by Friday the 1st of December.

We intend to supply a 15th December contract start date for the Phase 2 contracts, therefore to ensure we can move forward rapidly with the contracting work as soon as official confirmation has been supplied, it would be helpful if we could also receive answers to the following aspects of due diligence:

- If your company is under new ownership, or if a new company has been established, please provide details of the current ownership;
- If the IP portfolio relevant to the Phase 2 project has been updated, please send details of any new filings (we will require details of any relevant patent applications including filing or patent numbers as appropriate, license agreements with third parties etc.);
- Please provide copies of all sub-contractor quotes whose costings are included in the Phase 2 application.

If none of the above due diligence questions apply to your company or this project, please reply confirming this is the case for our records.

Please don't hesitate to get in touch if you have any queries.

Best regards,

Emily

From: **Emily C. Boyce** emily.boyce@hee.co.uk
Subject: RE: SBRI Healthcare: Phase 2 Provisional Award
Date: 14 December 2017 at 10:44
To: Laurence Pearce laurence@xim.ai
Cc: SBRIEnquiries SBRIEnquiries@hee.co.uk, Gina Pelletier gina@xim.ai

EB

Dear Laurence,

Thanks for looking over the schedules. We'd be happy to move forward to a final contract with your suggested changes.

I am also happy to confirm that NHS England has now ratified the Phase 2 award to Xim, therefore you should receive an official offer letter from SBRI Healthcare shortly.

With the due diligence information from Xim also received, I'm attaching a final version of the SBRI Healthcare Phase 2 contract. If you are satisfied with this, I'd be grateful if you could provide us with a signed scanned copy as well as posting two signed (wet ink) copies addressed the SBRI Healthcare team at the Health Enterprise East Ltd address below.

As soon as we have received the signed electronic copy, Xim can supply us with the project's Q1 invoice. Invoices should be made out to "Health Enterprise East", and include both Xim's VAT number and indicate the invoiced VAT component. Please send this invoice both to SBRIenquiries@hee.co.uk and accounts@hee.co.uk.

We tend to schedule a kick off telephone call in the first couple weeks of the contract's commencement date, which gives us the opportunity to outline the invoicing, reporting and PR requirements, as well as better appreciate the current status of the project and answer any questions you may have. Could you please let me know what times/dates from the 18th of December onwards might be convenient for this call?

Best wishes,
Emily

Emily Boyce PhD
Innovation Manager

Health Enterprise East Ltd
Milton Hall
Ely Road
Milton
Cambridge
CB24 6WZ

Tel: 01223 928 040
E-mail: emily.boyce@hee.co.uk
Web: www.hee.org.uk

SBRI HEALTHCARE
medtech ACCELERATOR
A Health Enterprise East Joint Venture
APPLY NOW!
