# Supplementary material for: Developing the Accuracy of Vital Sign Measurements Using the Lifelight Software Application in Comparison to Standard of Care Methods: Observational Study Protocol
Source: JMIR Res Protoc. 2021 Jan 28;10(1):e14326. doi: 10.2196/14326 (PMC7878110; doi:10.2196/14326)
Supplement: Multimedia Appendix 2 [file resprot_v10i1e14326_app2.doc]

**
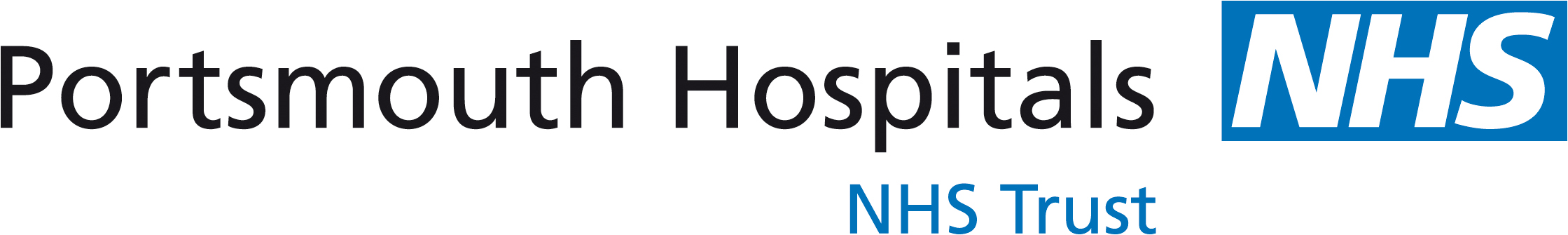
**

Research Office

1st Floor, Gloucester House

Queen Alexandra Hospital

Cosham

Portsmouth

PO6 3LY

Tel: 023 9228 6236

Fax: 023 9228 6037

Email: research.office@porthosp.nhs.uk

#### DESIGN REVIEW FORM

Front sheet details are confidential

| - Name of Reviewer: | **Dr Thomas Brown** |
| --- | --- |
| - Institution/Organisation: | **Portsmouth Hospitals NHS Trust** |
| - Reviewer’s Signature: |  |

#### RESEARCH DESIGN REVIEW FORM

Thank you for agreeing to provide a Design Review of the enclosed research proposal on behalf of the Portsmouth Hospitals NHS Trust. Please complete your assessment on this form and qualify your views where possible. The R&D Office staff can be contacted at any time should you have questions or concerns about the peer review process. Please note that the R&D Office will arrange for a separate review of strategic relevance or ‘Topic Area’. Your assessment will be passed unattributed to the research team.

On completion of your review please sign the front sheet and return this form to the R&D Office.

| R&D Office Use Only  Project Details: | | **Dates:** | | |
| --- | --- | --- | --- | --- |
| Title: | VISION-D | Sent to Reviewer On: | |  |
| Local Ref No: | PHT/2018/25 | To be Returned By: | |  |
| Principle Investigator Details: | | **Enclosures:** | | |
| Name: | Dr Thomas Jones | Protocol: | V1 | |
| Institution: | Portsmouth Hospitals NHS Trust | C.V. Details: | ☑ | |
|  |  | Other: |  | |

#### RESEARCH DESIGN REVIEW FORM

Please provide your views on any aspect of the project proposal, commenting specifically on the numbered areas identified in the left-hand column. Please then provide a summary of your views in the table overleaf. Both assessments will be copied and passed, unattributed, to the lead researcher.

| 1. **Research Quality** | |
| --- | --- |
| Please give your opinion on the overall quality of the design. Prompts: **Background and literature:** Is the current state of knowledge outlined, well structured, coherent and well -referenced?  **Question:** Is there a clear research question/ purpose, which leads on from the background and literature?  **Objectives:** Are the objectives clearly stated, appropriate and achievable?  **Design:** Is there a rationale for the approach? Are the methods chosen suitable and appropriate? Is the sample population described with reasons given for sample size? Will the sample size provide meaningful data once analysed? Are the research tools (such as questionnaires /interviews) well structured, informed, and suitable for analysis?  **Analysis:** Is there an effective analytical plan? | The study design is overall of high quality and I have no concerns regarding the ability of this study to gather the necessary data.  The background of the protocol explains the current state, and limitations, of vital sign measurement. It makes the purpose of the study quite clear and provides the necessary setting for this research.  There is no research question, but this is not necessary given the objectives of the study. The objectives are clearly stated and should be met by the design of the study. The design is appropriate. The sample size is not fixed, but the reason for this is explained. The data collected will be used by the sponsor, so there is no data analysis as such within this part of the study. |

| 1. **Research Planning & Practice** | |
| --- | --- |
| Please give your opinion on the overall research planning and practice.  **Prompts:**  **Project Management:** Is there evidence of a well-structured and achievable plan? Is the timetable realistic? Is the project manageable given the resources identified?  **The Investigative team:** Does the team have the appropriate multi-disciplinary and multi-professional mix to undertake the study? Are members of the team suitably qualified to carry out the stated methods and design? Would you advise further collaboration to ensure the study can be managed effectively? | The plan for the project is well structured. The timetable may be tight, but this is hard to predict given the uncertainty around the required sample size. The project should be manageable with current resources.  The investigative team have a good track record and appropriate mix of staff to conduct this study well. No further collaboration should be required. |

Please enter an assessment for each of the criteria listed below by ticking the appropriate box. The confidence level box is provided so that you can indicate your confidence in assessing the review report against the specified criterion (H=High, M=Medium, L=Low).

| Assessment  Criterion | Supported | Supported with Specific changes * | Not Supported | Confidence Level H/M/L |
| --- | --- | --- | --- | --- |
| 1. Research Quality | ☑ |  |  |  |
| 2. Research Planning & Practice | ☑ |  |  |  |

| 1. Overall assessment | ☑ |  |  |  |
| --- | --- | --- | --- | --- |

*** Please document any specific changes required, overleaf**

| Any General Comments/Overall Assessment Please clearly state any **specific changes** you would require to the protocol/research team  No changes required. |
| --- |

Thank you for taking the time to complete this design review on behalf of Portsmouth Hospitals NHS Trust.

Please sign the front sheet and return with enclosures to:

The Research Office

1st Floor, Gloucester House

Queen Alexandra Hospital

Cosham, PO6 3LY

Tel: 023 9228 6236

Fax: 02392 296037

Email: research.office@porthosp.nhs.uk
